# Certification information on trustworthy digital repository websites: A content analysis

**DOI:** 10.1371/journal.pone.0242525

**Published:** 2020-12-09

**Authors:** Devan Ray Donaldson

**Affiliations:** Department of Information and Library Science, Luddy School of Informatics, Computing, and Engineering, Indiana University, Bloomington, Indiana, United States of America; University of Cape Town, SOUTH AFRICA

## Abstract

In 1996, an international group of representatives from national archives and libraries, universities, industry, publishing offices, and other government and private sector organizations first articulated the need for certified Trustworthy Digital Repositories (TDRs). Henceforth, multiple standards for TDRs have developed worldwide and their reviewers provide third party audit of digital repositories. Even though hundreds of repositories are currently certified, we do not know if audit and certification of TDRs actually matters. For example, we do not know if digital repositories are actually better at preserving digital information after certification than they were before. Additionally, we do not know if TDRs preserve digital information better than their counterparts, although TDR standards definitely promulgate this assumption. One way of assessing whether audit and certification of TDRs matters is to study its impact on TDRs’ stakeholders (e.g., funders, data producers, data consumers). As an initial critical step forward, this study examines what certification-related information repositories actually include on their websites since repository websites provide a means of disseminating information. Using findings from a content analysis of 91 TDR-certified repository websites, this research examines: 1) written statements about TDR status, 2) the presence of TDR seals and their location, 3) whether the seals hyperlink to additional certification information, 4) the extent to which the certification process is explained, and 5) whether audit reports are shared. Nearly three-fourths of the repository websites provide TDR status statements and put seals in one or more places; nearly 60% post audit reports and link seals to additional certification information; and over one-third explain the certification process. Directions for future research and practical application of the results are discussed.

## Introduction

A trustworthy digital repository (TDR) is “one whose mission is to provide reliable, long-term access to managed digital resources to its designated community, now and in the future” [1, p. 5]. Nearly 30 years ago, an international group of representatives from national archives and libraries, colleges and universities, federal grant funding agencies, industry, publishing offices, and other government and private sector organizations first articulated the need for certified TDRs [2]. Since that time, multiple standards for TDRs have developed worldwide including, but not limited to:

- *Trustworthy Repositories Audit and Certification*: *Criteria and Checklist* (TRAC) [3] and its subsequent *Audit and Certification of Trustworthy Digital Repositories* (ISO 16363) [4]
- Catalogue of Criteria for Trusted Digital Repositories [5] and Seal [6] / *Criteria for Trustworthy Digital Archives* (DIN 31644) [7]
- International Council for Science (ICSU) World Data System (WDS), the *Data Seal of Approval* (DSA) [8], and their subsequents—the *Core Trustworthy Data Repositories Requirements* [9] and *CoreTrustSeal* [10]

At present, reviewers for these standards, such as the Center for Research Libraries (CRL) (for TRAC and ISO 16363), Primary Trustworthy Digital Repository Authorisation Body (PTAB) (for ISO 16363), Nestor Certification Working Group (for DIN 31644), and CoreTrustSeal Standards and Certification Board (for CoreTrustSeal), have audited and certified hundreds of digital repositories spanning five continents and over 26 countries.

The net result of certification is that it provides external validation that repositories which claim to preserve and provide access to digital resources both now and in the future are actually up to the challenge. That is, someone else has assessed your repository to determine that it can preserve, manage, and provide access to many types of digital materials in a variety of formats as well as curate those materials to enable search, discovery, and reuse with sufficient control for the digital material to be authentic, reliable, accessible and usable on a continuing basis. Consequently, all TDR standards include verbiage that touts the merits of certification to repositories’ various stakeholders (e.g., funders, data producers, depositors, and consumers). Specifically, these standards promise that TDR certification will make repositories’ stakeholders more confident that the data they contain will be protected, properly managed, and available for future reuse. As a visible token of successful certification, repositories are permitted to use seals and certification marks to communicate with stakeholders about their certification. However, little research examines what information, seals or otherwise, repositories use to communicate about their status as TDRs.

This paper investigates the use of repository websites as a means of sharing certification-related information and addresses the following research questions: 1) What information do repository websites provide about TDR certification status? And 2) Where is this information located?

## Background

### Audit and certification: Origins and development

The initial push for audit and certification of TDRs stemmed from concern about the fact that some important information in digital form that represents our cultural record had been lost forever. Examples include, but are not limited to: data from the 1960 census, the first email, and satellite observations of Brazil in the 1970s. Often technological obsolescence is the culprit in creating said data loss. The hardware and software that store and render the data become obsolete as newer (and in most cases better) hardware and software supersede them and the former cease to be produced. Digital preservation, ensuring access to digital content over time, requires an ongoing technical, legal, financial, and organizational commitment that not all institutions are able to handle. Waters and Garrett [2] first argued for audit and certification of TDRs to produce a network of repositories and identify institutions that could actually be trusted with the responsibility of preserving important digital information for the long term.

Building on the Waters and Garrett [2] report, the RLG/OCLC Working Group on Digital Archive Attributes [1] provided a formal definition for a TDR and articulated its attributes and responsibilities. TDR attributes include: compliance with the *Reference Model for an Open Archival Information System* (OAIS) [11, 12]; administrative responsibility; organizational viability; financial sustainability; technological and procedural suitability; system security; and procedural accountability. TDR responsibilities refer to both 1) high-level organizational and curatorial responsibilities as well as 2) operational responsibilities. Examples of high-level organizational and curatorial responsibilities include: defining the scope of collections in a repository; preservation and management of digital information throughout its lifecycle; addressing the needs of repositories’ various classes of stakeholders; ownership of materials and other legal issues. Examples of operational responsibilities include: negotiating with content providers; obtaining control of digital information; following documented policies and procedures; and adhering to best practice in creating digital resources. Additionally, TDRs’ operational responsibilities center on determining their designated community of users and ensuring that preserved information is understandable to those users.

Following from both the Waters and Garrett [2] and RLG/OCLC Working Group on Digital Archive Attributes [1] reports, the RLG-NARA Digital Repository Certification Task Force [3] produced criteria and a checklist for auditing and certifying TDRs. The criteria address three broad areas: 1) organizational infrastructure, 2) digital object management, 3) technologies, technical infrastructure, and security. Organizational infrastructure criteria focus on governance and organizational viability; organizational structure and staffing; procedural accountability and creating a policy framework; financial sustainability as well as contracts, licenses, and liabilities. Digital object management criteria emphasize the acquisition of content; the creation of archivable information; preservation planning; archival storage and preservation maintenance; information management; and access management. Technologies, technical infrastructure, and security criteria center on system infrastructure and use of appropriate technologies. The accompanying criteria checklist provides a tool for repository staff and auditors to record evidence in support of each criterion, including findings, observations, and results. The Center for Research Libraries (CRL) audited and certified the first six TDRs in the world using an earlier public draft of this standard better known as TRAC. The reports of these and other audits are publicly available.

Since 2007, several major developments have occurred regarding audit and certification of TDRs. First, TRAC was developed into an international standard, *Audit and Certification of Trustworthy Digital Repositories* (ISO 16363) [4], including the establishment of requirements for reviewers of candidate TDRs [13]. Second, a cluster of other TDR standards were developed. Some of these focus primarily on self-assessment, such as nestor’s *Catalogue of Criteria for Trusted Digital Repositories* and *Digital Repository Audit Method Based on Risk Assessment* (DRAMBORA) [14]. Other standards, such as the ICSU WDS standard for its regular members, DSA, and their subsequents, the *Core Trustworthy Data Repositories Requirements* and *CoreTrustSeal*, incorporate self-assessment into the audit process while emphasizing third-party review. For more information on the origins and development of repository audit and certification, see Digital Preservation Coalition [15] and Harvey and Weatherburn [16].

Results are mixed on the perceived value and usefulness of TDR standards. For example, McGovern [17, p. 333] states that TDR standards have helped “form the foundation for good practice in digital preservation.” In contrast, Seles [18] argues that TDR standards have too many assumptions embedded within them to apply or ever be useful to repositories in the developing world. For example, Seles found that TDR standards require use of resources and infrastructure that do not exist in several East African countries. Additionally, based on her sample of participants from developing countries, she found mixed acceptance of TDR standards by practitioners. For example, some of her participants utilized parts of a TDR standard modelling their digital repository operations against it, whereas others rejected the idea of certification, “arguing that it has no tangible benefit for their institutions generally or their repositories specifically” [18, pp. 277–278].

### The current landscape of TDRs

According to the European Framework for Audit and Certification of Digital Repositories, trustworthy digital repository certification occurs at three levels: 1) basic, now known as core, 2) extended, and 3) formal [19]. Core certification involves a self-audit which is externally reviewed by representatives from CoreTrustSeal. Repositories attain extended certification by successfully completing core certification along with a structured, externally reviewed and publicly available self-audit based on ISO 16363 or DIN 31644. Formal certification, the highest level of repository trustworthiness certification, involves a full audit and certification based on ISO 16363 or DIN 31644 in addition to surpassing core and extended certification levels.

Taken together, the lists of certified repositories on various standards’ websites show that hundreds of repositories currently have either core, extended, or formal certifications. In contrast, analysis of repository registries such as re3data.org indicate that there are thousands of uncertified repositories. Although the reasons for this disparity fall outside the scope of this paper, some researchers speculate that it is because most organizations do not require certification [20]. Other researchers have found that certification does not help practitioners address their operational problems, and they see little return in their investment in audit and certification for themselves or their institutions, all of which calls into question the usability and usefulness of TDR standards [18]. Notwithstanding, recent years have seen an uptake in the development and adoption of TDR standards, which in turn, has led to an increase in the number of certified repositories worldwide [21].

TDRs represent a broad range of institutions and domains. At formal and extended levels, TDRs include: community-supported archives, consortia of academic research libraries, digital libraries, collaborations between academic publishers and research libraries, cultural heritage organizations, national archives and libraries, institutes, and government publishing offices. At the core level, TDRs include: domain-specific data centers, archives, and repositories (e.g., World Data Service for Paleoclimatology, Strasbourg Astronomical Data Center, Roper Center for Public Opinion Research), national data centers, archives, and repositories (e.g., The Language Bank of Finland, Australian Data Archive), repositories based on data type (e.g., Survey Research Data Archive of Taiwan, Qualitative Data Repository), and commercial entities that store and make data publicly available (e.g., Mendeley Data). The examples above do not demonstrate the full range of TDRs; however, they offer insight into the current variety of TDRs. Also note that the categories above are not mutually exclusive.

### Audience matters when communicating about TDR certification

Those who manage or are otherwise responsible for digital repositories pursue TDR certification for a variety of reasons. For example, some staff want to identify the strengths and weaknesses of their repositories, or they want to improve their digital preservation policies and practices [22]. More often than not, however, decisions about audit and certification are inextricably linked to who repository staff want to communicate with about certification [23]. Analysis of certification standards’ documentation demonstrates that assessment results are intended for both internal and external audiences [24, 25]. In practice, research has shown that not only do repository staff communicate with their co-workers and upper management about certification internally, but they also communicate with funders, staff at other repositories, and to a lesser extent, users about certification externally.

Communication is necessary in cases where repositories’ funders require certification in order to receive or be eligible for additional funding. For example, both the Consortium of European Social Science Data Archives (CESSDA) and the Common Language Resources and Technology Infrastructure (CLARIN) require CoreTrustSeal certification for the repositories that they support financially. Additionally, research has shown that staff who seek funding for their repositories from other sources believe that including certification information in funding applications has a positive impact on their requests [26, 27].

Research has also shown that repository staff want staff at other repositories to know about their certification. Repository staff view TDR certification as an opportunity to “display” to their professional network of peers that they are committed “to following data curation standards and best practices through an external peer review process” [21, p. 8]. Additionally, repository staff seek certification because they think it communicates a similar level of professionalism and quality of their repositories to other repositories that have the same certification [26].

Research is inconclusive on the extent to which repository staff communicate with their users about certification. For example, in the aftermath of the establishment of the European Framework for Audit and Certification of Digital Repositories, representatives of Data Archiving and Networked Services (DANS) in The Netherlands reported mentioning their determination to be among the first officially certified digital archives to their users [22]. In other research, some staff reported that certification positively affected data consumers’ confidence in their repositories. Conversely, different repository staff either: 1) thought their users would not care, or they 2) thought certification might make a difference only if they did more to explain what it means [26]. According to Gladney [28], deciding whether or not to trust a TDR from a technical standpoint requires a great deal of knowledge and expertise that users are unlikely to have. Thus, explaining what repository trustworthiness means and what the certification process entails to users might not be worth the effort.

### TDRs and trust branding

Certifying TDRs has always been about trust branding. Most TDR standards include trust in the name (e.g., *Trustworthy* Repositories Audit and Certification (TRAC), Core*Trust*Seal, etc.). Certification by these standards suggests that a repository follows best practice in digital preservation, the assumption being that it can be trusted with preserving digital information for the long-term. In reality, we have no way of determining whether uncertified digital repositories are any better or worse able to preserve digital information than their certified counterparts. That is, unless we learn that some digital information has either been lost or deemed unrenderable.

Notwithstanding, TDR standards encourage transparency (e.g., making audit reports publicly available, etc.) and often permit the use of seals or certification marks for those who have successfully passed a third-party audit. This approach is not new. For over a century, governments, non-profit organizations, and companies have utilized a wide variety of third-party seals to build trust with consumers in the areas of, for example, health, nutrition, environmental impact, and more recently internet security and e-commerce [29, 30]. Research has shown that in order for a third-party seal to positively affect a consumer, the consumer must be familiar with the brand; have knowledge and understanding of the third-party and seal; and third-party seals must be important to the product or service category and the individual consumer [31]. Applied to TDRs, these findings suggest that when stakeholders are familiar with a repository, having knowledge and understanding of the seal and the third party that certified it, then the seals may positively affect their trust. However, before we can begin examining the impact of TDR seals and certification marks, we must first determine whether repositories who have become certified actually use them. In early tests of third-party certification for the DSA, for example, some repository staff mentioned not utilizing the seals to promote their archives because the certification process was not yet formalized and they still needed to pass all of the requirements of the standard [22]. Now that a greater number of repositories are certified by a variety of third parties, it is both timely and appropriate to assess repositories’ display of certification information, including their utilization of TDR certification marks and seals.

## Methods

To address the research questions, I performed a content analysis of certified repository websites based on steps and procedures outlined in Herring [32] and Krippendorf [33].

### Repository website selection

To include repositories from all three certification levels (e.g., core, extended, and formal), I selected the websites of repositories that were certified by CoreTrustSeal (e.g., core certification), NestorSeal (e.g., extended certification), CRL (e.g., formal certification), and PTAB (e.g., formal certification) for this study. I excluded repositories that only had DSA and/or WDS certifications as CoreTrustSeal supersedes both of those certification programs and is therefore the most up-to-date standard.

In April 2020, I downloaded the complete lists of current certified repositories from the CoreTrustSeal (n = 81), NestorSeal (n = 4), CRL (n = 6), and PTAB (n = 2) websites to establish the study sample (n = 93). Of the 93 certifications, two repositories, DANS Easy and ZBW, had more than one certification; both had CoreTrustSeal and nestorSeal certifications. Thus, I examined 91 repository websites for this study.

### Definition of categories and codes

I developed a codebook for this study based on a review of the literature on TDRs, information from TDR standards websites, and visual inspection of a random sample of eight repository websites, two from each TDR standard’s list of certified repositories (see Table 1).

**Table 1:** Codebook.

Categories	Codes
TDR Status Statements	1) No TDR Status Statement
	2) TDR Status Statement on One Subsidiary Page Only
	3) TDR Status Statement on Multiple Pages (except homepage)
	4) TDR Status Statement on Multiple Pages (homepage included)
Explanation of Repository Trustworthiness	1) No Explanation of Repository Trustworthiness on Website
	2) Written Explanation of Repository Trustworthiness on Website
Seal Presence	1) No Seal Posted
	2) Seal on Homepage Only
	3) Seal on One Subsidiary Page Only
	4) Seal on Multiple Pages
Seal Location	1) No seal anywhere
	2) Seal near top or in header
	3) Seal in middle or main body
	4) Seal near bottom or in footer
	5) Seal to the left of page
	6) Seal to the right of page
	7) Seal in multiple places and pages
Seal Hyperlinks	1) Not applicable (no seal)
	2) Seal Not Clickable
	3) Seal Hyperlinks to Standard’s Website
	4) Seal Hyperlinks to Audit Results
	5) Seal Hyperlinks to Additional Certification Information on Repository Website
Audit Results	1) No Audit Reports Posted or Linked to
	2) Final Report Posted or Linked to Only
	3) Self-Audit and Final Report Posted or Linked to

### Coder training, coding, and interrater reliability

Coder training involved six steps. First, to train in using the codebook, I coded the content of a random sample of one-fifth (n = 19) of the websites that were selected for the study. To identify as much certification-related information as possible for coding, I visually inspected all the websites’ homepages and subsidiary pages (i.e., about sections, archiving and preservation sections, certification sections, news sections, etc.). Then, I typed the name of the TDR standard as well as the terms Trustworthy Digital Repository, Trustworthy, Certification, and Certified in each website’s search bar to find any additional certification-related information. As a final precaution, I included the name of each repository along with the search terms described above as Google searches to identify any additional information from the site that the websites’ search engines may have omitted.

Second, I met with a second-year LIS graduate student with research experience to discuss the codebook and two examples of repository websites that I had coded. Third, the graduate student independently coded three websites from the list of websites that I had coded. Fourth, we compared our codes for the three websites, discussing any differences and resolving any disagreements. Fifth, the graduate student coded the remainder of the sample independently. Finally, I calculated Cohen’s kappa to assess interrater reliability for the codes that we both gave for the same sample of repository websites. Cohen’s kappa was k = 0.72 indicating 72% agreement in coding between us.

I coded the remaining 74 websites entering all of the data in a password-protected spreadsheet using Google Sheets, a free, web-based software spreadsheet program offered by Google.

### Data analysis and interpretation

Using various commands in Google Sheets, I calculated descriptive statistics for each of the categories and codes for all 91 websites. Altogether, I created six pivot tables utilizing the count function to assess the frequency of use of codes for each category (i.e., variable) listed in the codebook.

## Findings

Repositories can provide a wide range of information about TDR certification on their websites. Examples of this information include: attestations of their certification, explanations of the certification process, seals and certification marks, hyperlinks, and audit reports. However, repositories vary greatly in which type or combinations of certification-related information they actually present via their websites. This section reports on the different types of information that 91 repository websites provide regarding TDR certification.

### TDR status statements

A majority of repositories (n = 68 or 73%) provide written statements about their TDR status on their websites. Places where these statements are found include: 1) homepages, 2) news/press releases or blogs, and 3) subsidiary pages/sections (e.g., about sections; preservation, archiving, or curation sections; certification sections; TDR sections; services sections; data sections; etc.).

Repositories vary regarding where they place their TDR status statements and how many different places they put them (see Table 2). Thirty-five repositories put their TDR status statements in more than one place on their websites. For example, by including the statement “QDR has been certified as compliant with CoreTrustSeal standards” in its footer, the Qualitative Data Repository (QDR) at Syracuse University posts its TDR status statement on every page of its website. As another example, ZBW Leibniz-Informationszentrum Wirtschaft provides statements about being certified as reliable and trustworthy with the nestor seal on its history and long-term digital archiving web pages.

**Table 2:** The number of certified repositories that post TDR status statements on their websites, including their location(s).

Third Party Auditor	No TDR Status Statement	TDR Status Statement on One Subsidiary Page Only	TDR Status Statement on Multiple Pages (except homepage)	TDR Status Statement on Multiple Pages (homepage included)	Total (Per Third Party Auditor)
CRL			5	1	6
CTS Reviewers	24	31	18	8	81
Nestor Reviewers		2	2		4
PTAB	1		1		2
Total (Across Third Party Auditors)	25	33	26	9	93

Thirty-three repositories put their TDR status statements in one place only. For example, in the “Confidence in LDC Language Resources” page, the Linguistic Data Consortium (LDC) repository states that it is recognized as a trustworthy data repository under the CoreTrustSeal certification.

Twenty-five repositories do not include any TDR status statements anywhere on their websites.

### The certification process

In contrast to the provision of TDR status statements on repository websites, just over one-third (n = 32) of repositories provide explanations of the certification process on their websites (see Table 3). For example, the news release entitled, “ISSDA awarded the CoreTrustSeal 2017–2019” posted on the Irish Social Science Data Archive (ISSDA) website explains the CoreTrustSeal certification process in detail:

The CoreTrustSeal is a core level certification based on the DSA-WDS Core Trustworthy Data Repositories Requirements catalogue and procedures. This universal catalogue of requirements reflects the core characteristics of trustworthy data repositories. Core certification involves a minimally intensive process whereby data repositories supply evidence that they are sustainable and trustworthy. A repository first conducts an internal self-assessment, which is then reviewed by community peers. Such assessments help data communities—producers, repositories, and consumers—to improve the quality and transparency of their processes, and to increase awareness of and compliance with established standards. CoreTrustSeal is a community based non-profit organization promoting sustainable and trustworthy data infrastructures.

**Table 3:** Explanation of repository trustworthiness on TDR websites.

Third Party Auditor	No Explanation of Repository Trustworthiness on Website	Written Explanation of Repository Trustworthiness on Website	Total (Per Third Party Auditor)
CRL	1	5	6
CTS Reviewers	58	23	81
Nestor Reviewers	1	3	4
PTAB	1	1	2
Total (Across Third Party Auditors)	61	32	93

### The presence and location of seals

Repositories vary regarding whether they actually post the TDR seals that they have earned on their websites (see Table 4). Nearly half of the repositories (n = 45 or 48%) put TDR seals on multiple pages of their websites. For example, by including the CoreTrustSeal in its header, the Research data portal FDAT posts the CoreTrustSeal at the top of multiple pages. As another example, the Technische Informationsbibliothek Hanover (TIB) posts the nestor seal in a news release and its digital preservation section.

**Table 4:** The number of certified repositories that post TDR seals on their websites, including their location(s).

Third Party Auditor	No Seal Posted	Seal on Homepage Only	Seal on One Subsidiary Page Only	Seal on Multiple Pages	Total (Per Third Party Auditor)
CRL*	6				6
CTS Reviewers	13	18	7	43	81
Nestor Reviewers			2	2	4
PTAB	2				2
Total (Across Third Party Auditors)	21	18	9	45	93

*As of the time of this study, CRL did not confer seals or certification marks.

Twenty-seven repositories put their TDR seals in one place only. For example, the Digital Repository of Ireland posts the CoreTrustSeal in its About page. As another example, TalkBank posts the CoreTrustSeal on its homepage.

The 66 repositories that post seals or certification marks on one or more web pages put them in a variety of different places on any given page (see Table 5). Choices on where repositories place these seals include: at the top or in the header; in the middle or main body; at the bottom or in the footer; on the left side; on the right side; in multiple places on the same page; and in multiple places on multiple pages. For example, DataverseNO posts the CoreTrustSeal in both the header and footer of its homepage. As another example, the Strasbourg Astronomical Data Center includes the CoreTrustSeal as part of a featured news feed item in the main body of its homepage and in the footer.

**Table 5:** The location of seals on any given page of a certified repository website.

Third Party Auditor	No seal anywhere	Seal in top or header	Seal in middle or main body	Seal in bottom or footer	Seal to the left of page	Seal to the right of page	Seal in multiple places and pages	Total (Per Third Party Auditor)
CRL*	6							6
CTS Reviewers	13	6	18	32	2	1	9	81
Nestor Reviewers			2	1		1		4
PTAB	2							2
Total (Across Third Party Auditors)	21	6	20	33	2	2	9	93

*As of the time of this study, CRL did not confer seals or certification marks.

Twenty-one repositories do not post seals or certification marks anywhere. Part of the reason for this is that CRL does not confer seals or certification marks; thus, repositories that are certified by them (n = 6) do not have seals to denote their certification. Additionally, the IMS repository’s CoreTrustSeal was not visible because it had a broken image.

### Seal hyperlinks

Sixty-four percent (n = 58) of the repositories posted seals with hyperlinks in their websites (see Table 6). Of these, 31 repositories include seals that hyperlink to audit report results which contain details about how reviewers assessed the repositories’ compliance with each TDR criterion. For example, the CoreTrustSeal posted in the footer of the World Data Center—Sunspot Index and Long-term Solar Observations (SILSO) website hyperlinks to an assessment of SILSO’s adherence to the Core Trustworthy Data Repository Requirements.

**Table 6:** The number of certified repositories that post TDR seals with hyperlinks to various locations.

Third Party Auditor	N/A, No Seal	Not Clickable	Hyperlink to Standard’s Website	Hyperlink to Audit Results	Hyperlink to Add’l Cert. Info. on Repo. Website	Total (Per Third Party Auditor)
CRL*	6					6
CTS Reviewers	13	10	21	31	5	80
Nestor Reviewers		2			1	3
PTAB	2					2
Total (Across Third Party Auditors)	21	12	21	31	6	91

*As of the time of this study, CRL did not confer seals or certification marks.

Six repositories include seals with hyperlinks to other parts of the repositories’ websites that contain additional certification information. Different hyperlinks include: certification pages; pages within the repository websites devoted specifically to the standards; and data stewardship pages. For example, the CoreTrustSeal on the Inter-university Consortium for Political and Social Research (ICPSR) homepage hyperlinks to a data stewardship page stating that ICPSR is a CoreTrustSeal core certified repository.

Twenty-one include seals with hyperlinks to standards’ websites. Different hyperlinks include links to standards’: homepages; synopsis and about pages; lists of certified repositories; and requirements. For example, the CoreTrustSeal in the About section of the National Snow and Ice Data Center (NSIDC) hyperlinks to a list of certified repositories on the CoreTrustSeal website.

I found two anomalies. First, the CoreTrustSeal on the World Data Center—Renewable Resources and Environment website hyperlinks back to the World Data Center—Renewable Resources and Environment website homepage. Second, the nestor seal posted on the “Digital Preservation” section of the Technische Informationsbibliothek Hanover (TIB) website hyperlinks to a page on the nestor website that no longer exists which generates a 404 error.

### Audit reports

Just over half of the repositories post or provide links to self-audit or final audit reports on their websites (see Table 7). For example, the certification section of the Deutsche Nationalbibliothek website makes both the nestor seal self-audit and final audit report available for download.

**Table 7:** The number of certified repositories that post or provide links to self-audits or final audit reports.

Third Party Auditor	No Audit Reports Posted	Final Report Only	Self-Audit and Final Report	Total (Per Third Party Auditor)
CRL	1	2	3	6
CTS Reviewers	37	44		81
Nestor Reviewers		2	2	4
PTAB	2			2
Total (Across Third Party Auditors)	40	48	5	93

## Discussion

Prior to this study, there was no systematic, empirical investigation of what, if any, information repositories put on websites regarding their TDR status. This study’s findings provide key insights into what information repositories actually provide including its placement.

The findings from this study could be used as a starting point for a new set of research studies assessing the potential impact of certification information on TDRs’ stakeholders. Specifically, now that I have identified the various types of certification-related information that repositories actually use on their websites, we can begin to assess their impact. For example, a repository can assess whether providing no information (control group), one piece of information (e.g., a statement that the repository is a TDR), two pieces of information (e.g., a statement that the repository is a TDR and a seal or certification mark), or three pieces of information (e.g., a statement that the repository is a TDR, a seal or certification mark, and a link to audit report results) affects stakeholders’ trust in the repository. By including different classes of stakeholders in such studies (e.g., repository staff at other institutions, upper management, funders, users, etc.), repositories can also assess whether the impact of providing different levels of certification information varies by stakeholder type. More broadly, a study with multiple repositories can assess the impact of providing the same types of certification information on stakeholders from multiple repositories. Such studies could illuminate whether what works for one repository’s stakeholders works for another’s. Separate studies could assess whether where certification information is placed affects stakeholders’ identification of it as well as its impact. While other studies could examine whether the frequency with which certification information is included on TDRs’ websites plays a role in the same.

Since prior research suggests that a consumer must be familiar with a brand; have knowledge and understanding of the third-party and seal; and third-party seals must be important to the product or service category and the individual consumer for a third-party seal to be effective [31], we need research on how each of these factors or combinations of factors actually affect different classes of TDRs’ stakeholders. Based on findings from prior research (e.g., [26]), I speculate that different classes of stakeholders have varying knowledge, understanding, and regard for the importance of TDR certification. For example, repository staff at other TDRs are more likely to have knowledge of TDR certification than funders or users. Going forward, adequate measures of stakeholders’ knowledge, understanding, and perceived importance of TDR certification and seals need to be developed and validated so that we may empirically test these and other assumptions in both the short- and long-term as more repositories become certified. In the studies described above, such measures would be useful for creating profiles of stakeholder types and comparing them.

For TDR stakeholders who are unfamiliar with TDR standards and/or do not understand their importance, new research could explore how to increase: their familiarity with TDRs as a brand, their knowledge and understanding of the third parties that certify TDRs and the seals that denote certification; and the importance of TDR seals and certification marks to the digital preservation service industry and its individual stakeholders. I suspect that all three of these areas require more attention and scientific inquiry now where there is an increase in TDR certifications particularly at the core level. As a start, the 32 websites that I found included information about the certification process could be used for a future study since that information may increase stakeholders’ knowledge and understanding of TDR certification.

Studies such as all the ones described above are important not only because the certification process is “a conversation between the repository and its stakeholders,” but so is its aftermath [23, p. 125].

In this study, just under a quarter of repositories did not post any seals or certification marks on their websites. Part of the reason for this was because, as of the time of this study, CRL did not have a seal or certification mark for the six repositories that they certified to use. For the remaining 15 repositories that were certified by other standards, why did they choose not to post seals or certification marks on their websites despite having earned the right to do so? Perhaps these repositories use other means besides their websites to communicate with relevant stakeholders about certification. In any case, future research should explore this issue.

## Conclusion

TDR standards are examples of frameworks for conceptualizing and framing digital preservation problems; “the frameworks are useful as tools only to the extent they help do the work” [34, p. 80]. Research has shown that repositories can effectively preserve digital information without necessarily following best practices; in fact, those who try to follow specific best practices often struggle to do so, including TDRs [35]. In reality, we do not know if audit and certification of TDRs actually matters. For example, we do not know if digital repositories are actually better at preserving digital information after certification than they were before. Additionally, we do not know if TDRs preserve digital information better than their counterparts, although TDR standards definitely promulgate this assumption.

One way of assessing whether audit and certification of TDRs matters is to examine its impact on TDRs’ stakeholders. By examining what certification-related information repositories actually include on their websites, this study lays the groundwork for important future work on this topic.

## Supporting information

S1 Dataset (ODS)
